# Distinct patterns of parental involvement in Chinese families and preschoolers’ social adjustment

**DOI:** 10.3389/fpsyg.2025.1616138

**Published:** 2025-09-01

**Authors:** Zhongling Wu, Dianyue Zhang, Lichun Chen, Liang Chen, Wenxin Zhang

**Affiliations:** ^1^Faculty of Education, Shandong Normal University, Jinan, China; ^2^Faculty of Education, University of Macau, Taipa, Macau SAR, China; ^3^Faculty of Psychology, Shandong Normal University, Jinan, China; ^4^Shandong Provincial Key Laboratory of Brain Science and Mental Health, Jinan, China

**Keywords:** parental involvement, maternal involvement, preschool, latent profile analysis, social development

## Abstract

**Introduction:**

Parental involvement during early childhood is crucial for promoting children’s social development. However, few studies have explored both maternal and paternal involvement and their combined effects on children’s social adjustment.

**Methods:**

The person-centered approach was conducted to examine the profiles of maternal and paternal involvement in a sample of 535 Chinese families with preschool children. Furthermore, the present study investigated the associations between these parental involvement profiles and preschoolers’ social adjustment.

**Results and conclusion:**

Latent profile analysis identified a total of four profiles of parental involvement: *Moderate maternal-low paternal involvement* profile (21.9%), *Both moderate involvement* profile (38.1%), *High maternal-moderate paternal involvement* profile (28.2%), and *Both high involvement profile* (11.8%). Children in *Both high involvement* and *High maternal-moderate paternal involvement* profiles exhibited higher social skills compared to those in lower-involvement profiles. These findings enhance our understanding of home-based parental involvement patterns and their associations with preschoolers’ social development.

## 1 Introduction

The family environment serves as a critical proximal microsystem that significantly influences children’s cognitive and social development. Within the family environment, parental involvement has been demonstrated to promote children’s physical and mental well-being, socioemotional adjustment, behavioral outcomes, and academic performance (Ahmetoglu et al., 2022; Fantuzzo et al., 2004). Therefore, contemporary early childhood education and intervention initiatives emphasize family involvement as a key driver of child development (Hughes-Scholes and Gavidia-Payne, 2019). Although several studies have examined the role of parental involvement for children’s development (Hickey et al., 2020; Kawabata et al., 2011), relatively few studies have simultaneously focused on both maternal and paternal involvement and explored their combined effects on children’s adjustment (Rodríguez Ruíz et al., 2019). Furthermore, existing research frequently treats home-based involvement as an undifferentiated construct, neglecting its multidimensional nature (e.g., socialization, didactic, caregiving, and physical play domains) (Yotyodying and Wild, 2016). Therefore, the present study examined comprehensive patterns of parental involvement in Chinese families with preschool-aged children. Parental involvement has been demonstrated to significantly impact various domains of children’s social adjustment, including social skills, externalizing behaviors, and internalizing problems (Ahmetoglu et al., 2022). Considering children’s social adjustment has a rapid development, the role of parents during this period is particularly crucial (Rose et al., 2012). Therefore, this study aimed to investigate the associations between distinct patterns of parental involvement and social outcomes among preschool children.

### 1.1 Parental home-based involvement

Parental involvement refers to a construct including diverse parental involvement practices, such as home-based involvement, school-based involvement and home-school communication (Liu et al., 2023). Among these dimensions, home-based involvement (e.g., daily routines, learning activities, and visiting the library) plays a significant role in children’s social development (Fantuzzo et al., 2004). Furthermore, neurodevelopmental research has proved that supportive family experiences enhance neural connectivity (Arslan et al., 2011; Gerhardt, 2006). Given these neurobiological and psychological considerations, it is essential to investigate the characteristics of home-based involvement for preschool children.

Regarding home-based involvement during preschool years, Cabrera et al. (2004) identified four distinct subtypes of involvement activities including socialization, didactic, caregiving, and physical play, which help comprehensively understand parents’ family involvement. Socialization activity refers to parents’ involvement in activities connecting with the external world (e.g., participation in community or social events) (Ahmetoglu et al., 2022). Didactic activity involves parents’ involvement in children’s environmental exploration and learning through observation and imitation (Jia and Yu, 2021). Caregiving activity comprises routine-oriented tasks such as feeding and physical care. Physical play includes play that involves physical activity. This classification enables a clearer understanding of the different roles parents play in children’s development.

Substantial research has been conducted in understanding the distinct roles of maternal and paternal involvement in multiple developmental outcomes. Empirical studies consistently indicate that mothers are involved more in basic caregiving and routine-oriented tasks (Cano et al., 2019), whereas fathers are less involved in basic childcare and engage more frequently in play and didactic activities (Bornstein et al., 2016). Nevertheless, a critical gap persists in the literature, as most studies approach maternal and paternal involvement as separate constructs, thereby neglecting their joint effects within family systems. The lack of integrated research limits our understanding of potential synergistic effects that may arise from combined parental involvement patterns (Cabrera et al., 2014). Consequently, it is necessary to adopt a family systems perspective to investigate how mothers and fathers collaboratively coordinate their involvement to influence children’s developmental outcomes.

### 1.2 Distinct parental involvement patterns in families and their relation to child development

Extensive research has investigated the impact of family involvement on child developmental outcomes (Fantuzzo et al., 2004; Raikes et al., 2006). However, researchers always consider family involvement either as a unitary phenomenon or as separate dimensions using variable-oriented approaches (Castro et al., 2004; Fantuzzo et al., 2013). While such analytical strategies effectively demonstrate between-person differences, they are limited in revealing within-person differences across various aspects of family involvement (Collins and Lanza, 2010). In contrast, person-oriented methodologies overcome this limitation by classifying families into different subgroups (Collins and Lanza, 2010). Therefore, using the person-centered approach is essential to uncover meaningful patterns of family involvement, allowing for more tailored and effective interventions that account for the unique dynamics within different families.

There is limited evidence regarding patterns of home-based involvement using person-centered approaches. For example, Bulotsky-Shearer et al. (2012) identified six distinct profiles in home and classroom experiences among Head Start children. The most common profile (47.5%) was about low levels of parental involvement at home and school, coupled with average classroom quality. The other five profiles were “Low parent school & home involvement, very low classroom quality profile” (8.8%), “High parent school involvement, very low classroom quality” (5.0% of the sample), “High parent home involvement, very low classroom quality” (2.0%), “High parent school involvement, above average classroom quality” (24.0%) and “High parent school & home involvement, above average classroom quality” (12.6%). Regarding the associations between profiles and child development, the findings showed that children with the lowest levels of parent involvement and classroom quality had the worst academic performance and social development (Bulotsky-Shearer et al., 2012).

McWayne et al. (2016) identified different family involvement patterns among low-income Latino families. Three profiles were identified among Spanish-speaking families: “the Low Engagement group” (31%), “the Average Engagement group” (51%), and “the High Supplemental Education and School Participation group” (18%). Among the English-speaking sample, “the High Engagement group” comprised 48% of the sample, “the Average Engagement group” comprised 44% and “the Low Engagement group” comprised only 8%. Notably, the analysis revealed that higher engagement levels had a positive association with improved language and social skills among children in both language subgroups.

In a more recent study, Jeon et al. (2020) explored family engagement in two types of Early Head Start programs. Their person-centered analyses revealed three distinct profiles for home-based programs: Profile 1 “Low home involvement and relationship” (12%), Profile 2 “High home and low specific program involvement” (69%), and Profile 3 “High home and specific program involvement” (19%). Results indicated that children in Profile 2 and Profile 3 demonstrated significantly better engagement/orientation than those in Profile 1. The analysis also found three patterns for center-based programs, including Profile 1 “Low home and high program involvement” (8%), Profile 2 “High home and low program involvement” (50%) and Profile 3 “High involvement” (42%). Findings from center-based program revealed that children in Profiles 1 and 3 exhibited fewer behavioral problems than those in Profile 2, and children in Profile 3 showed superior social competence compared to children in Profile 2.

Emerging research has specifically examined patterns of father involvement in a sample of low-income young children (Yoon et al., 2021). Results of latent class analysis yielded four profiles of father involvement: “high positive involvement” (48%), “engaged but harsh discipline” (42%), “low cognitive stimulation” (8%), and “lower involvement” (2%). The low cognitive stimulation profile was related to more problem behaviors and less socioemotional and cognitive development.

Synthesis of existing literature, current evidence has primarily examined family involvement in conjunction with classroom or school participation, focused on low-income populations, or analyzed variations across Early Head Start programs. However, no empirical study has systematically explored the distinct patterns of family involvement activities as an independent research focus. From the perspective of family systems theory, the parental involvement in the family environment constitutes the most proximal and influential context for child development. Given the significance of parental involvement in home activities, the person-centered approach was used in the present study to identify distinct family involvement profiles that provide a comprehensive framework for understanding family dynamics in early childhood.

### 1.3 Mother and father involvement practices in Chinese families

Sociocultural values fundamentally shape children’s developmental contexts through culturally-embedded parental involvement in daily caregiving and interactions (Vélez-Agosto et al., 2017). In contemporary Chinese families, traditional gender roles, characterized by the notion that “men govern external matters while women oversee domestic affairs” (男主外女主内), have historically positioned mothers as primary caregivers and fathers as primary breadwinners (Li, 2020). However, sociocultural transformations since the late 20th century have changed the traditional norms. In 2022, Chinese women constituted 43.2% of China’s total workforce (National Bureau of Statistics of China, 2023). This demographic shift indicated that more and more mothers had dual roles in caregiving and employment (Zhou, 2017). Concurrently, the traditional roles of fathers have changed. Fathers had invested more time in participating in daily caregiving activities (Du et al., 2018). In addition, research demonstrates that Chinese fathers, much like fathers in Western counterparts, prefer engaging in activities that foster positive and stimulating parent–child interactions over routine caregiving tasks (Doucet, 2015). These findings reflect a gradual but significant transformation marked by growing paternal engagement in Chinese society. Despite empirical advances, research on maternal and paternal involvement dynamics in Chinese families remains underdeveloped. Existing studies have primarily examined parental roles in isolation, failing to capture how mothers and fathers collaboratively influence child development within the family system. Future investigations should prioritize examining the interactive effects of maternal and paternal involvement, with particular emphasis on China’s unique sociocultural context.

### 1.4 The present study

Research on the latent profiles of family involvement that incorporate both maternal and paternal contributions and their associations with children’s adjustment in two-parent families is particularly lacking. The study aimed to explore patterns of parental involvement and examine how these patterns influence preschool-age children’s socioemotional adjustment in Chinese families. Specifically, we will compare the similarities and differences between paternal and maternal involvement across each dimension. In addition, we will cross-classify parents’ involvement profiles to identify distinct patterns. Given the limited research on home-based parental involvement, the specific number of parental involvement profiles was not hypothesized. Finally, we will examine how these profiles relate to children’s social adjustment. Based on existing findings (e.g., Jeon et al., 2020; Yoon et al., 2021), we hypothesize that children exposed to high levels of parental involvement will exhibit the most favorable social adjustment.

## 2 Methods

### 2.1 Participants

The study was conducted in two preschools in Shandong Province, China. Both mothers and fathers of 535 preschool children participated in this study. Children’s ages ranged from 41.04 to 77.04 months, with an average age of 60.72 months (*SD* = 0.52). Approximately 44.9% of the children were girls. Mothers’ ages ranged from 23 to 50 years (*M* = 35.96, *SD* = 5.35), while fathers’ ages ranged from 26 to 60 years (*M* = 37.24, *SD* = 5.67). Approximately 73.9% of the mothers and 68.4% of the fathers held at least a bachelor’s degree, 86% of the mothers and 89% of the fathers were employed in technical, semiprofessional, or professional jobs, and 10.7% of households reported a monthly income exceeding 16,000 RMB (see Table 1).

**Table 1:** Demographic information of the sample (*N* = 535).

Variables	*M* (*SD*)/%
Child age (year)	5.06 (0.52)
Child gender	
Boys	55.1%
Girls	44.9%
Family monthly income (scored from 1 to 7)	
1. <7000RMB	17.3%
2. 7000–8000RMB	9.9%
3. 8000–10000RMB	23.0%
4. 10000–12000RMB	21.3%
5. 12000–14000RMB	12.1%
6. 14000–16000RMB	5.6%
7. >16000RMB	10.7%

	Mother	Father
Parental age (year)	35.96(5.35)	37.24(5.67)
Parental education (scored from 1 to 6)		
1. Elementary school or below	0.6%	0.2%
2. Junior high school (including non-graduates)	2.4%	2.2%
3. High school (including non-graduates)	8.8%	7.9%
4. Junior college degree	14.4%	21.3%
5. Bachelor’s degree	61.9%	56.1%
6. Master’s degree	12.0%	12.3%
Parental occupational prestige (scored from 1 to 5)		
1. Temporary workers, unemployed, unemployable, unskilled and agricultural laborers.	7.7%	1.9%
2. Manual laborers and self-employed, skilled and equivalent workers.	6.4%	9.2%
3. General management and general professional and technical staff, clerical staff.	19.3%	18.1%
4. Middle-level managers and middle-level professional and technical personnel.	58.5%	47.7%
5. High-level management staff or administrators.	8.2%	23.2%

### 2.2 Procedures

In the initial phase of the research, parental consent was secured, which included permission for both the parents’ and their children’s participation. Consent forms were distributed to participating families through classroom teachers. The data collection process began with the administration of an online questionnaire, with parents receiving an invitation message containing a link to the online survey, along with clear instructions on how to complete it. Both mothers and fathers provided individual reports on their involvement in home-based activities and filled out questionnaires regarding demographic details. Mothers also reported children’s social skills, effortful control and problem behaviors. The online format was convenient for parents to fill out when they had time, which allowed for efficient data collection. Additionally, children were given small gifts, such as picture books or notebooks. The procedures were approved by the Institutional Review Board of the first author’s institution.

### 2.3 Measures

#### 2.3.1 Parental involvement

Maternal and paternal home-based involvement were measured using the Activities with Child Scale by mothers and fathers, respectively (Cabrera et al., 2004). The scale contains 31 items that evaluate four dimensions: Physical Play (e.g., “Play outside, a park or a playground with the child”), Didactic (e.g., “Singing nursery rhymes like ‘Twinkle Twinkle Little Star’ or ‘Little Swallow’ with the child”), Socialization (e.g., “Inviting relatives to visit and interact with the child”), and Caregiving (e.g., “Putting the child to bed”). The scale uses a 6-point rating system (“1 = Never” to “6 = Daily”), with higher scores indicating greater parental involvement. Following standardized translation and back-translation procedures, the scale was translated into Chinese by researchers specializing in developmental psychology. Confirmatory factor analysis (CFA) was employed to examine its validity in the Chinese context. Results showed that the model fit was acceptable (χ^2^/*df =* 3.21, CFI = 0.93, TLI = 0.92, RMSEA = 0.06, SRMR = 0.05). In this study, the Cronbach’s *α* coefficients for maternal physical play, didactic, socialization and caregiving were 0.93, 0.92, 0.95 and 0.84, respectively; the Cronbach’s α coefficients for paternal physical play, didactic, socialization and caregiving were 0.92, 0.94, 0.96 and 0.90, respectively.

#### 2.3.2 Social skills

Mothers completed the Social Skills Improvement System Rating Scale to assess their children’s social skills (SSIS-RS; Gresham and Elliott, 2008). The social skills scale consists of 46 items that assess a variety of social skills such as assertion, cooperation, empathy, and self-control. Mothers were asked to report how often children exhibited each described behavior (from “0 = Never” to “3 = Almost always”). This scale exhibits good psychometric properties in the Chinese context (Cheung et al., 2017; Wu et al., 2019). The Cronbach’s *α* coefficient for the social skills scale was 0.86 in this study.

#### 2.3.3 Problem behaviors

We used the mother-reported Child Behavior Checklist (CBCL; Achenbach, 1991) to assess children’s problem behaviors. The anxious/depressed subscale (e.g., “Difficulty in maintaining focused and persistent attention”) and the aggressive behavior subscale (e.g., “Frequently hits others”) were used in this study. Mothers provided objective reports on their children’s behaviors based on daily observations. The scale uses a 3-point scoring system ranging from “0 = Not true” to “2 = Very true,” with higher scores indicating more frequent problem behaviors. This scale exhibits good psychometric properties in the Chinese context (Wang et al., 2018). In this study, the Cronbach’s *α* coefficients for the anxious/depressed and aggressive behavior subscales were 0.84 and 0.93, respectively.

#### 2.3.4 Effortful control

Mothers completed the Child Behavior Questionnaire very short form to assess children’s effortful control (CBQ-VSF; Rothbart et al., 2001). The CBQ-VSF measures three dimensions: effortful control, negative affectivity, and surgency. The present study employed the 12-item effortful control scale (e.g., “When drawing or colouring in a book, shows strong concentration”). Mothers rated the applicability of each behavior to their child on a 7-point Likert scale. A higher score for each item reflects a higher level of a particular behavior. The CBQ-VSF has been successfully adapted into Chinese and exhibited good psychometric properties in the Chinese context (Fan et al., 2020). In the present study, the Cronbach’s α coefficient for the Effortful control subscale was 0.85.

### 2.4 Data analysis

First, paired samples *t*-tests were conducted to assess differences in home-based involvement between mothers and fathers within families, followed by repeated-measures ANOVAs to examine differences across the four home activities for mothers and fathers separately. Latent profile analysis (LPA) was then employed to categorize distinct patterns of parental home-based involvement through a model-fitting process. Beginning with a one-profile model, the number of profiles increased until there was no significant model fit improvement or additional profiles failed to provide more meaningful interpretation. To determine the optimal number of profiles, multiple fit indices were evaluated, including the Akaike information criterion (AIC), the Bayesian information criterion (BIC), the sample-size adjusted BIC (aBIC), the Lo–Mendell–Rubin likelihood ratio test (LMR-LRT), and entropy. Consistent with standard practice, the lower values of AIC, BIC, and aBIC indicated better model fit. The LMR-LRT was used to compare the fit of the specified model to a model with one less class. A statistically significant *p*-value suggested that the k-profile solution demonstrates significantly better model fit compared to its (k-1)-profile. Regarding classification accuracy, entropy values exceeding 0.80 were considered acceptable, with values approaching 1.00 indicating a better fit (Weller et al., 2020). Overall, the model with better values in AIC, BIC, aBIC, LMR-LRT, and entropy is preferred. To address the missing data, the Maximum likelihood estimation with nonnormality-robust standard errors (MLR in Mplus) was used. In addition, we considered the prevalence of each profile and the theoretical meaning or interpretability of the model when selecting the best model. Subsequently, multivariate analyses of covariance (MANOVAs) were conducted to investigate the associations between identified parental involvement profiles and the social outcomes of preschool children. The statistical software SPSS 21.0 and Mplus 8.7 were used to perform all these analyses in the present study.

## 3 Results

### 3.1 Common method bias testing

As the data on maternal involvement and the child’s social adjustment were all obtained by mothers, common method bias was assessed using Harman’s single-factor test (Podsakoff et al., 2003). The findings revealed that the variance explained by the first principal factor was 34.04% (<40%) (Tang and Wen, 2020), indicating that the results would not be affected by common method bias.

### 3.2 The differences in maternal and paternal involvement across various home activities

The descriptive statistics and correlation analysis across all study variables were presented in Table 2. To examine the differences in home-based activities between mothers and fathers within individual families, we conducted separate paired sample *t*-tests for Physical Play, Didactic, Socialization and Caregiving activities. The findings revealed statistically significant differences in the levels of all four activities involved by mothers compared to fathers. Notably, mothers demonstrated a higher degree of involvement than fathers, as indicated by the *t*-test results (*t*_Physical Play_ = 3.30, *p* < 0.001, Cohen’s *d* = 0.99; *t*_Didactic_ = 12.79, *p* < 0.001, Cohen’s *d* = 1.22; *t*_Socialization_ = 4.07, *p* < 0.001, Cohen’s *d* = 1.03; *t*_Caregiving_ = 8.64, *p* < 0.001, Cohen’s *d* = 1.23) (see Table 3).

**Table 2:** Bivariate correlations of the measuring variables.

Variables	1	2	3	4	5	6	7	8	9	10	11	12
1. Mother physical play												
2. Mother didactic	0.69^***^											
3. Mother socialization	0.67^***^	0.60^***^										
4. Mother caregiving	0.47^***^	0.56^***^	0.39^***^									
5. Father physical play	0.37^***^	0.26^***^	0.31^***^	0.11^**^								
6. Father didactic	0.31^***^	0.26^***^	0.27^***^	0.15^***^	0.76^***^							
7. Father socialization	0.35^***^	0.24^***^	0.38^***^	0.14^***^	0.79^***^	0.78^***^						
8. Father caregiving	0.25^***^	0.18^***^	0.26^***^	0.20^***^	0.64^***^	0.70^***^	0.63^***^					
9. Social skills	0.54^***^	0.54^***^	0.46^***^	0.27^***^	0.27^***^	0.25^***^	0.29^***^	0.17^***^				
10. Effortful control	0.31^***^	0.35^***^	0.23^***^	0.15^***^	0.17^***^	0.22^***^	0.18^***^	0.12^**^	0.47^***^			
11. Anxious depressed	−0.06	−0.05	0.07	0.03	0.02	0.01	0.02	0.07	−0.14^**^	−0.12^**^		
12. Aggressive behavior	−0.05	−0.04	0.05	0.01	0.05	0.04	0.04	0.09*	−0.14^***^	−0.12^**^	0.89^***^	
*M*	3.84	4.38	3.73	3.92	3.69	3.70	3.55	3.46	92.64	5.17	1.26	1.24
*SD*	0.86	0.96	0.94	0.95	0.90	1.04	0.91	0.99	23.15	0.84	0.34	0.33

^*^p < 0.05; ^**^p < 0.01; ^***^p < 0.001.

**Table 3:** Paired samples *t*-test results of the four dimensions of parental involvement.

Variables	Mother (*M* ± *SD*)	Father (*M* ± *SD*)	*t*	Cohen’s *d*
Physical play	3.84 ± 0.86	3.69 ± 0.90	3.30^***^	0.99
Didactic	4.38 ± 0.96	3.70 ± 1.04	12.79^***^	1.22
Socialization	3.73 ± 0.94	3.55 ± 0.91	4.07^***^	1.03
Caregiving	3.92 ± 0.95	3.46 ± 0.99	8.64^***^	1.23

^***^p < 0.001.

Repeated-measures ANOVAs were conducted to examine differences in parental involvement across four activities (Physical Play, Didactic, Socialization and Caregiving), separately for mothers and fathers. Results indicated significant differences across the four activities for mothers [*F* (2.63, 1402.12) = 113.43, *p* < 0.001, partial *η*^2^ = 0.18]. *Post hoc* comparisons revealed that mother’s Didactic (*M* = 4.38, *SD* = 0.96) was significantly higher than mother’s Physical Play (*M* = 3.84, *SD* = 0.86, *p* < 0.001), mother’s Socialization (*M* = 3.73, *SD* = 0.94, *p* < 0.001), and mother’s Caregiving (*M* = 3.92, *SD* = 0.95, *p* < 0.001). Mother’s Caregiving was higher than mother’s Socialization (*p* < 0.001), and mother’s Physical Play exceeded mother’s Socialization (*p* < 0.01). For fathers, significant differences were also observed across the four activities (*F* (2.69, 1437.70) = 27.70, *p* < 0.001, partial *η*^2^ = 0.05). Post-hoc tests revealed that father’s Physical Play (*M* = 3.69, *SD* = 0.90) was significantly higher than father’s Socialization (*M* = 3.55, *SD* = 0.91, *p* < 0.001) and father’s Caregiving (*M* = 3.46, *SD* = 0.99, *p* < 0.001). Father’s Didactic (*M* = 3.70, *SD* = 1.04) was also significantly higher than father’s Socialization (*p* < 0.001) and father’s Caregiving (*p* < 0.001). Additionally, father’s Socialization was higher than father’s Caregiving (*p* < 0.01) (see Table 4).

**Table 4:** Descriptive statistics and comparisons of the four profiles on each indicator variable.

Variables	Mother				Father			
	*M (SD)*	*F*	Partial *η*^2^	*Post-hoc*	*M (SD)*	*F*	Partial *η*^2^	*Post-hoc*
Physical play	3.84(0.86)	113.43^***^	0.18	2>1, 3, 4 4>3 1>3	3.69 (0.90)	27.70^***^	0.05	1, 2>3, 4 3>4
Didactic	4.38(0.96)				3.70 (1.04)			
Socialization	3.73(0.94)				3.55 (0.91)			
Caregiving	3.92(0.95)				3.46 (0.99)			

1, physical play; 2, didactic; 3, socialization; 4, caregiving; ^***^p < 0.001.

### 3.3 Identifying parental involvement patterns

The enumeration indices of the competing LPA models were presented in Table 5. The results showed that values of AIC, BIC, and aBIC gradually decreased from the 1-profile model to the 5-profile model, suggesting that each additional profile resulted in a better model fit. Meanwhile, the entropy values were acceptable (Weller et al., 2020), indicating clear classification of profiles (Celeux and Soromenho, 1996). Considering there was one profile with a relatively low percentage (7.5%, *n* = 40) of families in the 5-profile model, we excluded the solutions with five profiles. Although the LMR-LRT values were statistically non-significant between the 3-profile and 4-profile, the 4-profile model exhibited a lower BIC value, and the 4-profile model distinguished subcategories of parental involvement in a more fine-grained way compared to the 3-profile model. Specifically, the 3-profile model only captured heterogeneity in the degree of parental involvement, while the 4-profile model captured heterogeneity in the degree of both paternal and maternal involvement and their combinations. Meanwhile, the 4-profile model had a group size ratio of 11.8%, which is greater than the minimum group size ratio recognized by many researchers (Collins and Lanza, 2010). With all these considerations, the 4-profile model was identified as the final model that optimally balanced model parsimony with model interpretability compared to the other models.

**Table 5:** Model fit Indices from the latent profile analysis.

Model	AIC	BIC	a-BIC	Entropy	LMR-LRT *p*-value
1-profile	11676.52	11745.04	11694.25	-	-
2-profile	10767.59	10874.65	10795.29	0.92	0.00
3-profile	10288.41	10434.00	10326.08	0.87	0.01
4-profile	10074.79	10258.93	10122.44	0.80	0.22
5-profile	9905.79	10128.47	9963.40	0.83	0.35

To further understand the four identified profiles, MANOVA was conducted to assess how these profiles varied across the various home-based activities of the instrument. The effect sizes, denoted by the partial eta-squared (*η*^2^) values, were listed in Table 6. Results indicated that four profiles had statistically significant main effects on all home-based activities (i.e., Physical Play, Didactic, Socialization and Caregiving activities), providing evidence that the profiles were empirically grounded.

**Table 6:** Means differences in different home activities by four profiles.

Variables	Profile 1	Profile 2	Profile 3	Profile 4	*F*	partial *η*^2^
Mother physical play	3.32(0.62)^c^	3.43(0.58)^c^	4.34(0.69)^b^	4.89(0.82)^a^	136.08^***^	0.44
Mother didactic	3.85(0.81)^b^	3.83(0.67)^b^	5.13(0.61)^b^	5.29(0.81)^a^	154.52^***^	0.47
Mother socialization	3.13(0.75)^d^	3.36(0.65)^c^	4.31(0.77)^b^	4.67(0.95)^a^	106.08^***^	0.38
Mother caregiving	3.57(0.86)^d^	3.52(0.73)^b^	4.50(0.82)^a^	4.51(1.05)^a^	58.18^***^	0.25
Father physical play	2.74(0.58)^d^	3.89(0.57)^b^	3.55 (0.57)^c^	5.17 (0.60)^a^	255.92^***^	0.59
Father didactic	2.52 (0.67)^d^	3.99 (0.57)^b^	3.52 (0.67)^c^	5.44 (0.49)^a^	332.73^***^	0.65
Father socialization	2.62 (0.59)^d^	3.70 (0.57)^b^	3.40 (0.58)^c^	5.14 (0.60)^a^	265.30^***^	0.60
Father caregiving	2.56 (0.69)^d^	3.66 (0.75)^b^	3.27 (0.66)^c^	4.94 (0.89)^a^	153.79^***^	0.47

Descriptive statistics include mean (standard deviation); ^a,b,c^Bonferroni *post-hoc* contrasts with ^***^p < 0.001; Profile 1, *Moderate maternal-low paternal involvement* profile; Profile 2, *Both moderate involvement profile*; Profile 3, *High maternal-moderate paternal involvement* profile; Profile 4, *Both high involvement* profile.

**Figure 1:**
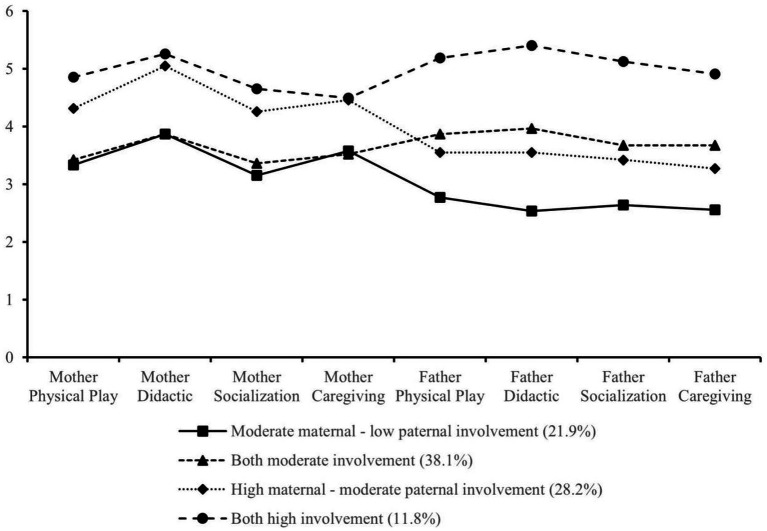
Parental involvement profile in Chinese families.

Profile 1 was characterized by moderate maternal involvement and low paternal involvement. We labeled this profile *Moderate maternal-low paternal involvement*, which represented about 21.9% (*n* = 117) of the sample. Profile 2, the largest subgroup with moderate maternal involvement and moderate paternal involvement, represented about 38.1% (*n* = 204) of the sample. This group was labeled as *Both moderate involvement*. Profile 3 showed high levels of maternal involvement and a relatively moderate level of paternal involvement, representing about 28.2% (*n* = 151) of the sample, which is named *High maternal-moderate paternal involvement*. Profile 4 exhibited overall high maternal and paternal involvement, with the smallest proportion of the sample represented at 11.8% (*n* = 63), and was labeled *Both high involvement* families (see Figure 1).

### 3.4 Associations between profiles of parental involvement and children’s social adjustment

To examine the link between parental involvement and children’s social adaptation, Multivariate Analysis of Covariance (MANCOVA) was employed, with control variables including gender, age and the family socio-economic status. The findings indicated a substantial multivariate effect for the home-based involvement profiles [Wilks’s lambda = 0.78, *F* (12, 1,310) = 10.65, *p* < 0.001]. Subsequent Bonferroni *post-hoc* comparisons revealed differences (see Table 7).

**Table 7:** Mean differences for child social adjustment variables by four profiles.

Variables	Profile 1	Profile 2	Profile 3	Profile 4	*F*	partial *η*^2^
Social skills	83.54 (22.92)^c^	86.58 (18.97)^c^	100.17 (21.25)^b^	113.24 (22.18)^a^	37.85^***^	0.19
Effortful control	4.90 (0.90)^b^	5.01 (0.77)^b^	5.40 (0.74)^a^	5.61 (0.87)^a^	15.96^***^	0.09
Anxious/ depressed	1.24 (0.26)	1.27 (0.33)	1.25 (0.33)	1.29 (0.48)	0.37	0.00
Aggressive behavior	1.22 (0.25)	1.24 (0.31)	1.22 (0.31)	1.30 (0.49)	1.15	0.01

Descriptive statistics include mean (standard deviation); ^a,b,c^Bonferroni *post-hoc* contrasts with ^***^p < 0.001; Profile 1, *Moderate maternal-low paternal involvement* profile; Profile 2, *Both moderate involvement* profile; Profile 3, *High maternal-moderate paternal involvement* profile; Profile 4, *Both high involvement* profile.

Based on analyses using the data in the table, children with *Both high involvement* profile (Profile 4) had significantly better social adaptation than the other three profiles, including showing higher social skills, and higher effortful control scores. By contrast, in terms of social skills and effortful control, the scores of *Moderate maternal-low paternal involvement* profile (Profile 1) and *Both Moderate involvement* profile (Profile 2) were relatively similar. Meanwhile, children with *High maternal-moderate paternal involvement* profile (Profile 3) had higher social skills and effortful control scores than those with *Both moderate involvement* profile (Profile 2) and *Moderate maternal-low paternal involvement* profile (Profile 1). There was no statistically significant mean difference in anxious/depression and aggressive behavior by profiles.

## 4 Discussion

This study explored the profiles of parental involvement in home-based activities and examined the associations between different profiles and children’s social adjustment. The findings revealed that four distinct profiles of maternal and paternal home-based involvement emerged in Chinese families. Moreover, the findings demonstrated that maternal and paternal home-based involvement profiles were significantly associated with children’s social skills but showed no significant associations with problem behaviors. Thus, these results contribute to the growing body of literature highlighting the importance of assessing both maternal and paternal involvement, as discussed in the following section.

Using the variable-centered approach, the present study first explored the differences between fathers’ and mothers’ involvement in various family activities. The results showed that in Chinese families, not only in caregiving activities, mothers demonstrated significantly more involvement than fathers across all measured activities with preschool children, including Physical Play, Didactic, Socialization and Caregiving activity. These findings align with existing literature indicating that mothers remain primary caregivers even in contemporary families (Bornstein et al., 2016). Consistent with traditional gender role expectations, men are expected to focus on paid work, while women take on more unpaid household and caregiving tasks (Hou et al., 2018). As a result, mothers are more involved in home activities than fathers.

This study found that both fathers and mothers spent much more time on didactic activities than on other activities. This is consistent with previous research that Chinese mothers are more focused on children’s academic achievements than Western mothers (Hoffman, 1988; Pearson and Rao, 2003). Chinese parents use numbers more frequently in communication with children to help develop children’s mathematical abilities (Chang et al., 2011). In China, knowledge-based examination is an important way to select talent. The traditional culture in China emphasize knowledge and early learning for young children. Consequently, parents tend to devote more time to cognitive educational activities with their young children (Luo et al., 2013).

Except for didactic activities, fathers were more involved in physical play than in the other two activities, while the least involved activity was caregiving activities. For mothers, the second most frequently engaged activity was caregiving activities, and the lowest involvement activity was physical activities at home. The findings are consistent with our expectation that mothers engage in more caregiving activities, while fathers engage in more physical activities, reflecting the role differentiation in Chinese culture rooted in the Confucian tradition. Fathers generally devote more time to play activities, whereas mothers typically spend more time on childcare activities (Bornstein et al., 2016). It is found that not only in the Chinese context, but also in other cultures, role differentiation between mothers and fathers is observed, with fathers always assuming the role of playmate to their children (Yaffe, 2023).

### 4.1 Observed parental home-based involvement profiles

The findings produced four profiles of family involvement, highlighting the heterogeneity in Chinese families’ involvement practices. Among these four profiles, the largest proportion of the sample (38.1%) was the *Both moderate involvement* profile in which both fathers and mothers had moderate levels of home involvement in all kinds of activities with young children. This consistency in parental involvement indicates that there are cooperative dynamics between mothers and fathers in contemporary Chinese families with preschool children.

The analysis revealed a congruent high-involvement profile (*Both high involvement profile* profile; 11.8% of the sample) characterized by uniformly high involvement levels from both parents across all measured family activities. Together with the moderate-involvement class, these two profiles collectively accounted for approximately half (49.9%) of the sample, indicating that a significant proportion of contemporary Chinese families exhibit synchronized parental involvement in early childhood rearing. Recent reports from Mainland China further support these findings, highlighting a shift in traditional gender roles in some families. One survey found that most Chinese fathers no longer adhere to conventional gender role boundaries in child-rearing, with 77% reporting they were involved in household chores and child-rearing activities just like mothers (Li, 2021; National Bureau of Statistics of China, 2001). Some researchers also found that Chinese fathers actively participated in traditionally maternal caregiving tasks, including feeding and bathing (Liong, 2017; Chuang and Su, 2009). These findings were consistent with Simons and Conger (2007) viewpoint, suggesting that parents tend to partner with those who share similar parenting styles. Through daily interactions like observational learning and experience sharing, their parenting approaches gradually converge, resulting in shared perspectives on childrearing.

Furthermore, nearly 50 % of families adopted inconsistent maternal and paternal involvement, represented by the *High maternal-moderate paternal involvement* profile (28.2%) and the *Moderate maternal-low paternal involvement* profile (21.9%). The *High maternal-moderate paternal involvement* profile was characterized by a higher level of maternal involvement and a moderate level of paternal involvement across the board. The *Moderate maternal-low paternal involvement* profile was about moderate maternal involvement and low paternal involvement as rated by both parents. Parental involvement in these two classes showed that mothers were more involved in all types of activities than fathers in the family. These two maternal-dominant profiles align with traditional Chinese gender norms, rooted in Confucian values, historically assigned mothers primary responsibility for childrearing while positioning fathers as distant authority figures and economic providers. In addition, the two maternal-dominant profiles (High-moderate and Moderate-low, totalling 50.1%) may be due to some mothers’ gatekeeping behaviors, where mothers consciously or unconsciously limit fathers’ involvement through criticism or rigid standards of care (Aytac and Schoppe-Sullivan, 2023).

### 4.2 Differences across profiles in children’s social development

In terms of the associations between involvement patterns and child developmental outcomes, findings showed that children in *Both high involvement* profile had significantly better social skills and higher effortful control scores than the other three profiles. Meanwhile, the children with the *High maternal-moderate paternal involvement* profile had higher social skills and effortful control scores than those with *Both moderate involvement* and *Moderate maternal-low paternal involvement* profiles.

Consistent with prior research (McWayne et al., 2016), the present results showed that parental involvement was positively associated with children’s social development during early childhood. Such involvement enables parents to better understand their children’s needs and to provide more effective support for children’s social skills development. Through daily interactions, children gain more opportunities to practice social skills. Additionally, parental involvement at home directly shapes children’s self-regulation skills. For instance, shared activities like reading aloud require children to practice self-regulatory behaviors, such as sitting still and maintaining attention (Froiland and Davison, 2016). In the early years, parents act as external regulators, and through their guidance, children gradually learn to self-regulate their behaviors and thoughts.

However, no significant differences in problem behaviors were observed across the four profiles of home involvement. This suggests that high levels of parental involvement across multiple dimensions may not necessarily reduce children’s behavioral problems. These results partially contradict previous studies linking low parental involvement with increased behavioral issues (McCormick et al., 2013). The observed discrepancies may stem from methodological differences in assessing involvement, variations in children’s age ranges, or differences in the developmental domains examined.

### 4.3 Implications for future research and practice

The interpretation of these findings should consider two key limitations of the study. First, as this research was conducted in a single province in China, caution should be taken when generalizing the results to other contexts. While Shandong Province shares common characteristics with most provinces in China, including rapid socioeconomic transformation and regional development disparities, it may differ from less developed regions, particularly in Central and Western China, in terms of educational and socioeconomic development indicators. Second, children’s social adjustment outcomes in this study were based on maternal reports. Hence, future research incorporating observational methods would enhance the systematic understanding of children’s social development and its associations with home-based involvement patterns. Such approaches could also yield more robust recommendations for family education practices.

Despite those limitations, the findings have implications for interventions. The results indicate that both maternal and paternal involvement in home-based activities present significant significance for promoting children’s social development, which needs to be considered simultaneously. Families with low involvement patterns are likely to obtain fewer developmental benefits from these interactions. Although this group represents a small portion of the population, these families may have greater risks or needs, requiring programs to provide targeted support to enhance their involvement. This focus is particularly important as research consistently shows that overall family involvement plays a critical role in children’s social development (Brand and Jungmann, 2014). From a clinical perspective, assessing both maternal and paternal involvement enables a more comprehensive understanding of family involvement patterns, supporting the creation of tailored intervention approaches. The present findings suggest that attempts to improve children’s social behaviors should include mothers as well as fathers, and that counsellors should address the needs of both parents. By establishing consistent involvement patterns through aligned communication and shared disciplinary approaches, mothers and fathers can create a unified supportive environment that optimally promotes their child’s social development.

## Data Availability

The original contributions presented in the study are included in the article/supplementary material, further inquiries can be directed to the corresponding author.
